# Comments on: Limitations of HPV DNA Testing in Screening of Cervical Adenocarcinomas

**DOI:** 10.1055/s-0039-1678591

**Published:** 2019-02

**Authors:** Marcos Emanuel de Alcântara Segura

**Affiliations:** 1 Lâmina Laboratory of Pathology and Cancer Prevention, Brasília, DF, Brazil

Dear Editor,

I read with great interest the article on HPV DNA testing for cervical cancer screening in Brazil, authored by the Brazilian Association for the Lower Genital Tract Pathology and Colposcopy (ABPTGIC, in the Portuguese acronym).^1^ In agreement with the authors, I have found irrefutable evidence sustaining the use of molecular detection of HPV DNA in this setting. However, every method, no matter how innovative or advanced, has limitations. The “Achilles' heel” of HPV DNA primary screening is the detection of glandular lesions. I found that the manuscript by the ABPTGIC tangentially addressed this issue.

An international cross-sectional study found that 38% of adenocarcinomas were not HPV-related.^2^ A recently proposed histologic classification of endocervical adenocarcinoma, known as International Endocervical Adenocarcinoma Criteria and Classification (IECC),^3^ uses the presence of HPV-related disease to histologically classify cervical adenocarcinomas. Among nonhuman papillomavirus-associated adenocarcinoma (NHPVA), the authors identify the following histological types: endometrial, gastric-type, minimal deviation, serous, clear-cell and mesonephric. In our practice, we have recently been involved in the case of an asymptomatic woman, HPV DNA-negative, who showed abnormal cytologic results interpreted as high-grade lesion. The patient proved to have a cervical clear-cell adenocarcinoma. This tumor is classified by the IECC group as a NHPVA and would not be identified in the screening scenario by molecular assays based on HPV DNA.

In summary, it is well recognized that HPV DNA detection delivers high sensibility, which is of great help, since cytologic-based screening is known to have low sensibility and high false-negative rates.^4^ ^5^ But the limitations of this molecular detection should be recognized, such as the great proportion of NHPVAs. If molecular testing for HPV DNA is to be used as a primary screening test, one must acknowledge that non-HPV-related tumors would be missed.

## Notes

Authors' Reply Dear Editor, The letter to the Editor “Limitations of HPV DNA testing in screening of cervical adenocarcinomas” mentioned the cross-sectional study of de Sanjose et al,^1^ who found that more than one third of invasive cervical cancer adenocarcinomas are HPV-negative. In this way, an HPV DNA-based screening program would be ineffective in detecting those lesions. We agree that screening with this test would have some limitations and maybe this was not properly highlighted in our paper. Screening as a public health program is designed to identify individuals at risk of developing a specific condition with high prevalence or health relevance. The vast majority of cervical cancers are HPV-positive squamous cell carcinomas, the main target of a cervical cancer screening program. The incidence of endocervical adenocarcinomas has been proportionally increasing due to expansion of screening, particularly in population-based programs.^2^ In the study by Stolnicu et al^3^, HPV-negative adenocarcinomas were diagnosed in more advanced stages than squamous carcinomas. Further studies are needed to clarify if this was due to later detection or a more aggressive behavior of the tumor. If the latter option was the case, screening would be less effective anyway. The strongest evidence about the efficacy of HPV DNA-based screening comes from Ronco et al^4^ in an extended follow-up of 4 randomized controlled trials with 176,464 women enrolled. The authors have found a greater protection against invasive cervical carcinoma using HPV DNA test compared with cytology. One of the most expressive results was the improvement in adenocarcinoma detection, showing that HPV DNA-based screening may improve the cytology-based programs deficiency in detecting glandular lesions.^5^
We recognize that one of the biggest challenges of cervical cancer screening is glandular lesions, especially because of its partially unknown natural history and probably less frequent association with HPV. More efforts should be addressed to improve the diagnosis of HPV-negative adenocarcinomas. However, considering the best available evidence until now, its effectiveness still allows us, as well as other authors and institutions, to recommend the use of these tests in cervical cancer screening in specific scenarios.
